# SATB1 preserves CD4^+^ T-cell fidelity and establishes Treg function in antitumor immunity

**DOI:** 10.26508/lsa.202603763

**Published:** 2026-07-14

**Authors:** Wooseok Seo, Chengcheng Zou, Kanako Shimizu, Ruka Setoguchi, Kiyokazu Kakugawa, Terumi Kohwi-Shigematsu, Shohei Hori, Shin-ichiro Fujii, Hiroyoshi Nishikawa, Ichiro Taniuchi

**Affiliations:** 1 Division of Cancer Immune Multicellular System Regulation, Center for Cancer Immunotherapy and Immunobiology (CCII), Kyoto University Graduate School of Medicine , Kyoto, Japan; 2 Department of Immunology, Nagoya University Graduate School of Medicine, Nagoya, Japan; 3 Laboratory for Transcriptional Regulation, RIKEN Center for Integrative Medical Sciences (IMS) , Yokohama, Japan; 4 Laboratory for Immunotherapy, RIKEN Center for Integrative Medical Sciences (IMS) , Yokohama, Japan; 5 Laboratory for Immunogenetics, RIKEN Center for Integrative Medical Sciences (IMS) , Yokohama, Japan; 6 Laboratory for Immune Crosstalk, RIKEN Center for Integrative Medical Sciences (IMS) , Yokohama, Japan; 7 Department of Orofacial Sciences, University of California , San Francisco, USA; 8 Laboratory of Immunology and Microbiology, Graduate School of Pharmaceutical Sciences, The University of Tokyo, Tokyo, Japan

## Abstract

The genome organizer special AT-rich sequence binding protein 1 (SATB1) is critical for the development of T lymphocyte subsets by regulating *Foxp3* and *Pdcd1* expression. However, its physiological role in mature peripheral T cells has remained obscured by the severe developmental defects caused by early-stage gene knockout models. Here, we used a *Thpok-cre* driver to specifically delete *Satb1* after CD4 lineage commitment. We demonstrate that SATB1 is continuously required to suppress *Foxp3* expression in conventional CD4^+^ T cells; however, this aberrant derepression does not confer Treg suppressive activity. In contrast, FoxP3^+^CD25^+^ Tregs isolated from SATB1-deficient mice exhibit functional defects in immune suppressive function. Although SATB1 deletion does not cause severe global defects in the Treg transcriptome, it disrupts essential FoxP3-mediated gene regulation, resulting in a targeted failure to silence transcripts detrimental to Treg fitness. Physiologically, this Treg-specific functional defect significantly enhances antitumor immunity in a melanoma model. Our findings reveal a dual requirement for SATB1, preserving lineage fidelity by repressing *Foxp3* in conventional T cells, while enforcing foundational programming required for Treg suppressive function.

## Abstract (precis)

SATB1 preserves CD4^+^ T-cell lineage fidelity by repressing *Foxp3* in conventional T cells and is essential for establishing FoxP3-mediated gene repression in regulatory T cells. Deleting SATB1 disrupts Treg suppressive function, unleashing potent antitumor immunity.

## Introduction

Special AT-rich sequence binding protein 1 (SATB1) is a crucial genome organizer that regulates T lymphocyte development and function (1, 2, 3). Its expression is highest in the thymus (4), where it plays a critical role in shaping the T-cell transcriptome. Deciphering the precise roles of SATB1 in mature peripheral T cells has been a long-standing challenge. Among its many targets are genes of profound clinical importance in cancer immunotherapy, the most notable of which is *Pdcd1* (5), which encodes the inhibitory receptor PD-1. SATB1 forms a transcriptional network on *Pdcd1* in CD8^+^ cytotoxic T cells to control T-cell activation and exhaustion. Another important target is *Foxp3* (6), which encodes the lineage-defining transcription factor FoxP3 for regulatory T cells (Tregs) by establishing super-enhancer regions (7, 8) and proper methylation (9).

The developmental complexity obscures SATB1’s role in a key immunological conundrum. Germline deletion of SATB1 is lethal on postnatal day 14–16 (10), and previous studies have relied on conditional knockout systems that delete the gene at early stages of T-cell development (e.g., using *Vav-cre* (11), *Lck-cre* (12), or *Cd4-cre* (13, 14, 15, 16, 17)). While these models established SATB1’s importance, they also introduced confounding variables, as the early loss of SATB1 severely disrupts fundamental developmental processes of T cells, including the lineage choice between CD4^+^ helper and CD8^+^ cytotoxic T cells (10, 13). This makes it difficult to determine whether observed phenotypes in mature T cells are a requirement for SATB1 in the periphery or are merely consequences of flawed development. An ideal model would therefore need to bypass the developmental effects to isolate SATB1’s function in mature T cells.

To address this question, using the *Thpok-cre* (18, 19) and *E8I-cre* (20, 21, 22, 23) is useful because target genes are specifically ablated in mature CD4^+^ or CD8^+^ single-positive thymocytes, respectively. Because the *Thpok-cre* driver targets the entire CD4^+^ T-cell compartment, it is better suited to examine SATB1’s role in the Tconv population, over a more lineage-restricted driver such as *Foxp3-cre*. Also, *Thpok-cre* becomes active from the stage of CD4-lineage commitment in thymocytes preceding the expression of *Foxp3*. This is important because Tregs become less dependent on SATB1 as they mature (6), suggesting that the SATB1-dependent Treg program might be established and stabilized before the full expression of FoxP3.

While recent studies have begun using this *Thpok-cre* model (6, 18, 19), the high-resolution functional and transcriptomic consequences within the mature CD4^+^ T-cell compartment have remained incompletely understood. Here, we leverage bulk and single-cell RNA sequencing alongside an in vivo tumor model to address the true functions of SATB1 in mature peripheral CD4^+^ T cells. This model allowed us to unmask the true functions of SATB1 in the mature peripheral CD4^+^ T-cell compartment. We show that the derepression of *Foxp3* by SATB1 deletion in mature CD4^+^ T cells does not confer suppressive function. Paradoxically, we find that *bona fide* FoxP3^+^CD25^+^ Tregs isolated from *Thpok-cre* mice require SATB1 for their function, as its loss renders them non-suppressive. We show that this defect is due to a failure of FoxP3-mediated gene repression and SATB1 is required for optimal FoxP3-mediated gene repression. We further show that this Treg-specific functional defect has significant in vivo consequences, as *Satb1*^*F/F*^; *Thpok-cre* mice exhibit enhanced antitumor immunity because of reduced functionality of Treg. Thus, our study using *Satb1^F/F^:Thpok-cre* mice unmasks a dual role for SATB1 in the mature CD4^+^ T-cell compartment, where it is required both to maintain Tconv lineage fidelity and to enforce the functional programming required for subsequent suppressive function in Tregs.

## Results

### SATB1 is required for the suppressive function of Tregs

**Figure 1.**
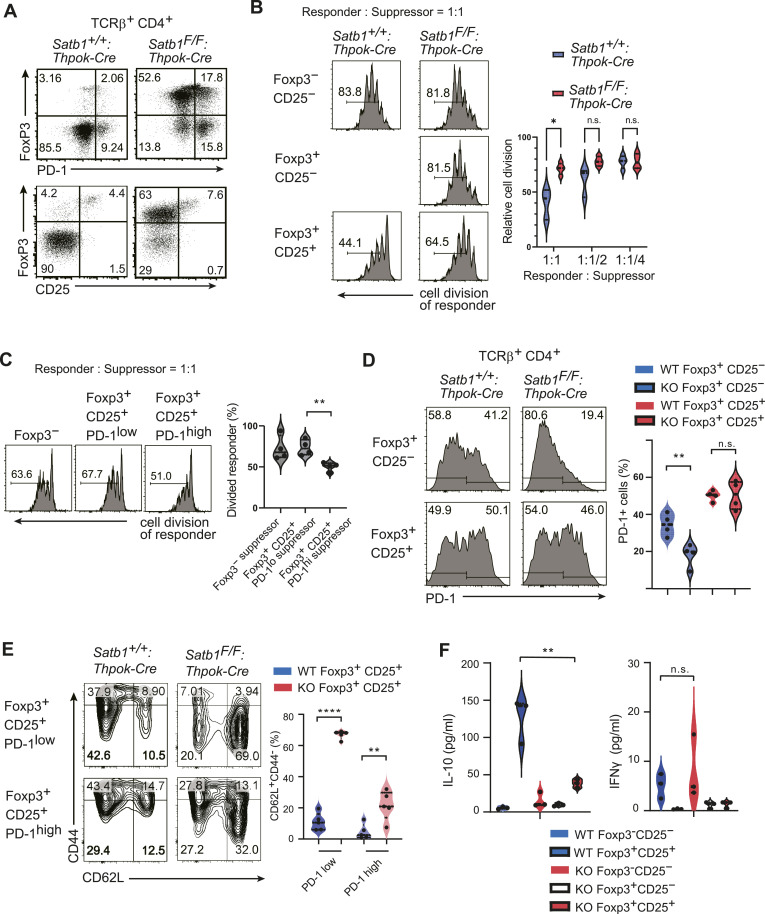
SATB1 is required for the suppressive function of Tregs. **(A)** Flow cytometric analysis of FoxP3 and PD-1 expression in resting nonactivated splenic CD4^+^ T cells from WT and *Satb1* conditional knockout mice. **(B)** Suppressive effects of CD4^+^FoxP3^+^CD25^+^, CD4^+^FoxP3^+^CD25^−^, or CD4^+^FoxP3^−^CD25^−^ population from WT (left) and *Satb1*^*F/F*^:*Thpok-cre* mice (right) on the proliferation of responder CD4^+^ T cells from WT mice were assessed by flow cytometry. The suppressor and responder cell ratios were tested with 1:1, 1:1/2, and 1:1/4. A summary graph of duplicates from two mice is shown on the right. **(C)** Suppressive effects of CD4^+^FoxP3^+^CD25^+^PD-1^high^ and CD4^+^FoxP3^+^CD25^+^PD-1^low^ population from WT mice on the proliferation of responder CD8^+^ T cells from WT mice were assessed by flow cytometry. The suppressor and responder cell ratios were tested with 1:1. A summary graph of the four independent assays shown on the right. **(D)** CD4^+^FoxP3^+^CD25^−^ (upper) and CD4^+^FoxP3^+^CD25^+^(lower) population from WT (left) and *Satb1*^*F/F*^:*Thpok-cre* mice (right) are examined for PD-1 expression. A representative result from three independent experiments is shown. **(E)** CD44 and CD62L expression was examined by flow cytometry from CD4^+^FoxP3^+^CD25^+^ CD25^+^PD-1^low^ population or CD4^+^FoxP3^+^CD25^+^PD-1^high^ of WT and *Satb1*^*F/F*^:*Thpok-cre* mice. A representative result from three independent experiments is shown. **(F)** Cytokines IL-10 and IFNγ were measured through Luminex assays from in vitro cultured cells of indicated genotypes. A summary graph of duplicates from two independent assays is shown on the right. Statistical significance is measured via unpaired two-tailed *t* tests and is presented as follows: n.s. nonsignificant, *P < 0.05, **P < 0.01, ****P < 0.0001.

Early-stage models such as *Lck-cre* and *Cd4-cre* cause severe developmental defects, whereas late-stage *Thpok-cre* and *Foxp3-cre* models have not been comprehensively examined for transcriptomic and functional changes within the CD4^+^ compartment. Because the *Thpok-cre* driver deletes *Satb1* after CD4 lineage commitment but before FoxP3 expression, we reasoned that this model can specifically interrogate the early pioneering steps of SATB1-mediated genome organization. We first tested whether SATB1 continues to repress its known targets in this post-commitment CD4^+^ compartment that includes conventional CD4^+^ T cells and Tregs. We found that *Satb1* deletion via *Thpok-cre* resulted in significant derepression of *Pdcd1* from splenic CD4^+^ T cells (Fig 1A upper), in addition to the previously observed derepression of *Foxp3* (Fig 1A below). It should be noted that the aberrant expression of *Pdcd1* was observed on nonactivated resting splenic CD4^+^ T cells. This was different from CD8^+^ T cells, in which *Pdcd1* was shown to be derepressed only in activated cells (5). This suggests that *Pdcd1* might be in an “always-on” state without SATB1, which normally acts as an “off switch” that is removed upon TCR engagement.

Because the functionality of SATB1-deficient Tregs was not directly examined before, we isolated these cells from spleens for an in vitro suppression assay. Using a *Foxp3-ires-gfp* reporter strain (24) to purify the cell populations, we found that the FoxP3^+^CD25^−^ cells did not show any suppressive activity against responder cells and behaved similarly to FoxP3^−^CD25^−^ non-Treg cells (Fig 1B). This indicates that the aberrant expression of FoxP3 alone does not confer regulatory function. Interestingly, SATB1-deficient FoxP3^+^CD25^+^ cells also exhibited significantly reduced suppressive activity compared with their SATB1-sufficient counterparts, indicating that bona fide Tregs do not function properly in the absence of SATB1.

The suppressive function of Tregs has been correlated with high PD-1 expression in previous literature (25, 26). Consistently, our in vitro suppression assay also showed that PD-1^high^ Treg cells, not PD-1^low^, had suppressive activity (Fig 1C). Therefore, it was possible that SATB1-deficient Tregs have less suppressive activity because of the changes in PD1 expression levels. Even though the SATB1-deficient FoxP3^+^CD25^−^ population expressed very low levels of PD-1, the SATB1-deficient FoxP3^+^CD25^+^ population exhibited PD-1 expression at a similar level to SATB1-sufficient populations (Fig 1D). This result suggests that PD-1 expression levels were not the cause of the reduced suppressive activity of SATB1-deficient Tregs. Instead, we found that SATB1-deficient FoxP3^+^CD25^+^ Treg cells had reduced CD44 expression compared with WT cells, indicating that they were in a less activated state (Fig 1E). Functionally, SATB1-deficient Tregs produced significantly lower levels of the key regulatory cytokine IL-10, whereas IFNγ production in non-Treg helper T cells was unchanged (Fig 1F). Collectively, our data indicate that in the absence of SATB1, Tregs have inherently reduced functionality in a PD-1–independent manner.

### SATB1 is required for optimal FoxP3-mediated gene repression

**Figure 2.**
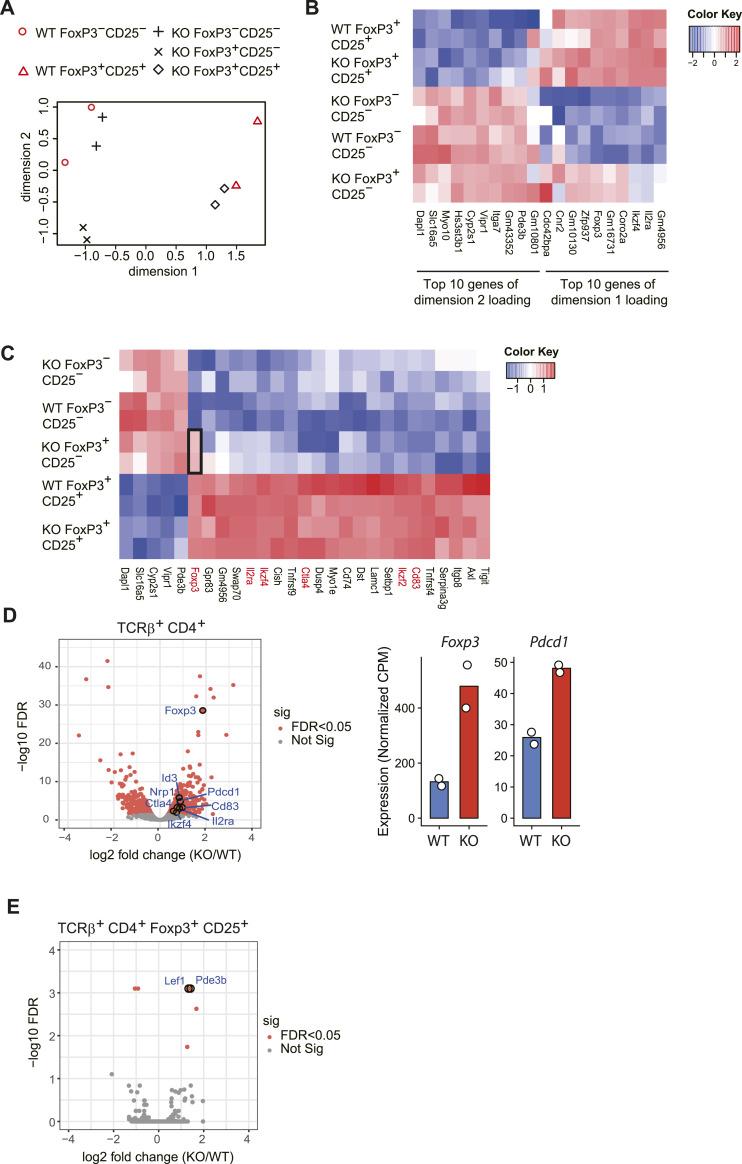
SATB1 functions as an essential co-factor for FoxP3-mediated gene repression. **(A)** Principal component analysis plot of RNA-seq from spleen CD4^+^ T cells from indicated populations of WT and *Satb1*^*F/F*^:*Thpok-cre* mice is shown as a replicate (n = 2). **(B)** Heatmap detailing the expression of the top 10 genes driving dimension 1 loading and the top 10 genes driving dimension two loading. **(C)** The RNA-seq result of FoxP3^+^CD25^−^ population from *Satb1*^*F/F*^:*Thpok-cre* mice were examined against the top 30 differentially expressed genes of the lowest *P*-values between WT FoxP3^+^CD25^+^ versus FoxP3^−^CD25^−^. **(D)** Volcano plot of RNA-seq result of CD4^+^ populations from WT and *Satb1*^*F/F*^:*Thpok-cre* mice as a replicate (n = 2) is shown (left). Selected genes are indicated by text labels. Normalized expression (counts per million) of *Pdcd1* and *Foxp3* of splenic CD4^+^ T cells from WT and *Satb1* cKO mice (right). Bars represent the mean, with dots indicating individual biological replicates (n = 2). **(E)** Volcano plot of RNA-seq result of CD4^+^FoxP3^+^CD25^+^ populations from WT and *Satb1*^*F/F*^:*Thpok-cre* mice as a replicate (n = 2) is shown. Selected genes are indicated by text labels.

To understand the molecular basis of this compromised functionality of SATB1-deficient Tregs isolated from spleens of *Satb1*^*F/F*^:*Thpok-cre* mice, we performed RNA-seq analyses on sorted CD4^+^ T-cell populations. Principal component analysis revealed that the aberrant SATB1-deficient FoxP3^+^CD25^−^ cells form an independent group (Fig 2A). However, an analysis of the axes indicates that this separation is highly dependent on dimension 1, which serves as the primary transcriptomic axis differentiating the Treg and non-Treg lineages (Fig 2B). Along this defining dimension 1 axis, the KO FoxP3^+^CD25^−^ population firmly segregates with the conventional non-Treg populations instead of Treg. Accordingly, a comparison of the top 10 genes driving dimension 1 loading confirms that the aberrant FoxP3^+^CD25^−^ population shares a core transcriptomic resemblance with non-Tregs. The independent clustering of these aberrant cells is instead driven primarily by genes under dimension 2, reflecting the isolated strong expression of *Foxp3* (Fig 2C) and a limited subset of genes rather than a global lineage shift.

To assess whether this signature represented canonical FoxP3 activity, we cross-referenced the top 100 drivers of dimension 2 against established direct FoxP3 targets (27). We observed an overlap of only 11 genes with no statistical significance (*P* = 0.65), indicating that the separation along dimension 2 is not the result of FoxP3 successfully engaging its canonical regulatory program, but rather reflects a dysregulated transcriptional state arising in the absence of SATB1’s genome-organizing capacity. This definitively supports our finding that *Foxp3* derepression alone fails to cross the primary transcriptomic threshold required to induce the full Treg signature program from conventional CD4^+^ T cells.

**Figure S1.**
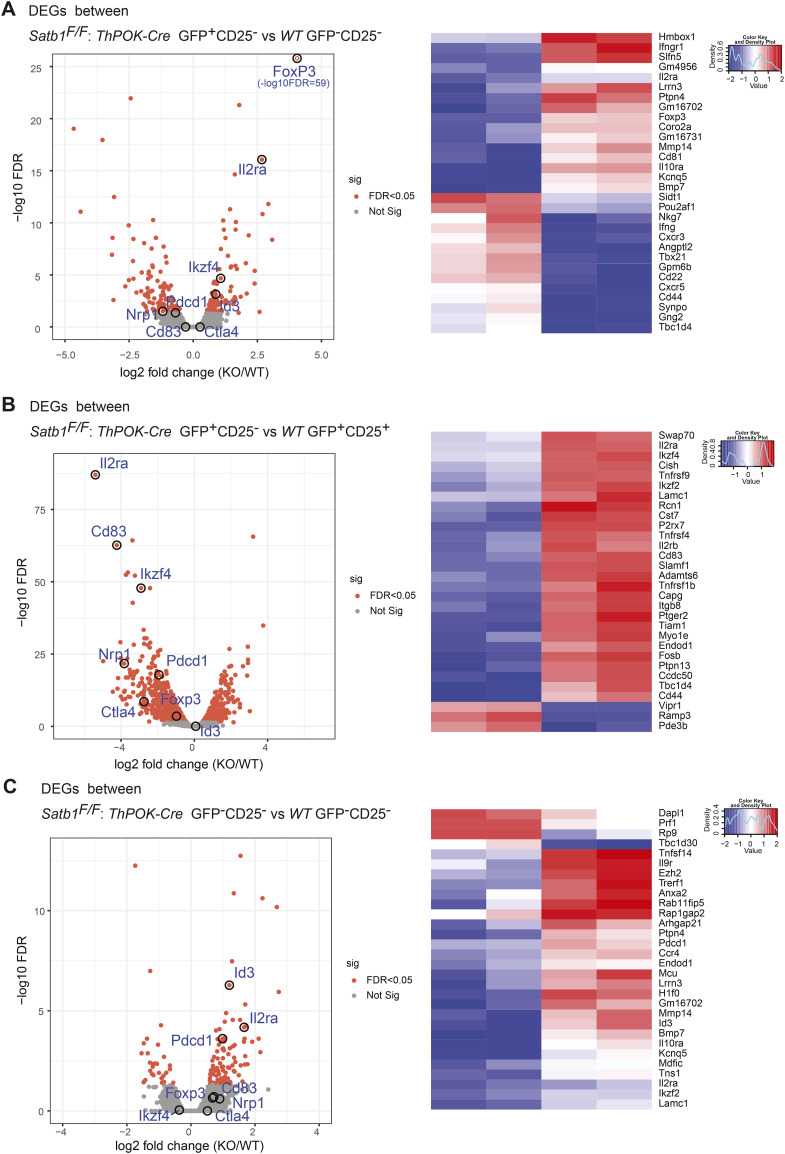
Transcriptomic analysis of differentially expressed genes driven by Satb1 deletion. **(A)** Volcano plot and accompanying heatmap showing DEGs when comparing the aberrant Satb1 knockout GFP^+^CD25^−^ population directly to WT GFP^−^CD25^−^ non-Tregs, highlighting that Foxp3 is massively up-regulated. **(B)** Volcano plot and accompanying heatmap comparing the aberrant knockout population against true WT GFP^+^CD25^+^ Tregs, revealing profound deficits in the expression of classical Treg signature markers (Il2ra, Cd83, and Nrp1). **(C)** Volcano plot and accompanying heatmap analyzing the strict non-Treg compartment between the knockout and WT (GFP^−^CD25^−^ versus GFP^−^CD25^−^), confirming that Satb1 deletion directly drives the up-regulation of Pdcd1 while leaving typical Treg markers completely unperturbed. DEGs, differentially expressed genes.

Further examination of these differentially expressed genes underscored this dichotomy (Fig S1). When comparing the aberrant *Satb1* knockout GFP^+^CD25^−^ population directly to WT GFP^−^CD25^−^ non-Tregs, *Foxp3* was massively up-regulated (Fig S1A). Conversely, comparing this aberrant knockout population against true WT GFP^+^CD25^+^ Tregs revealed profound deficits in the expression of classical Treg signature markers (*Il2ra*, *Cd83*, and *Nrp1*), confirming their transcriptomic failure to differentiate into Tregs despite the presence of *Foxp3* (Fig S1B). Moreover, analyzing the strict non-Treg compartment between the knockout and WT (GFP^−^CD25^−^ versus GFP^−^CD25^−^) confirmed that *Satb1* deletion directly drives the up-regulation of *Pdcd1*, while leaving typical Treg markers completely unperturbed (Fig S1C).

In the gene expression profiles of splenic CD4^+^ T cells, approximately 200 transcripts were differentially regulated by the loss of Satb1 with *Thpok-cre* from peripheral CD4^+^ T cells (Fig 2D). A gene ontology (GO) analysis of these transcripts did not yield significantly enriched programs. We note that this bulk RNA-seq analysis was performed with duplicate samples (n = 2); consequently, the statistical power may be limited, and increased replicates could potentially surface additional differentially expressed genes and pathways. Nevertheless, the current data suggest that Satb1 deficiency induces selective changes in specific target genes rather than global transcriptomic changes in peripheral CD4^+^ T cells. Consistent with previous findings (6, 19), *Foxp3* and *Pdcd1* were highly derepressed in splenic CD4^+^ T cells in the absence of SATB1, whereas other signature genes of Treg cells did not show significant differences, indicating that the transcriptomic differences in this unseparated population largely reflect the compositional shift driven by the emergence of nonfunctional FoxP3^+^ cells. To isolate true cell-intrinsic transcriptional changes from this macroscopic compositional shift, we compared strictly FACS-sorted subpopulations. Notably, when we compared SATB1-deficient FoxP3^+^CD25^+^ Tregs to their WT counterparts, only six genes were differentially expressed (Fig 2E). Among these, *Lef1* and *Pde3b* showed the highest statistical confidence. Both are known to be repressed by FoxP3, and maintaining their low expression is critical for proper Treg function (28, 29, 30, 31, 32). Therefore, our transcriptomic analysis proposes a model in which SATB1-deficient Tregs appear to have functional defects due to the failure to control the transcription of highly targeted genes such as *Lef1* and *Pde3b*.

### Absence of *Satb1* from mature CD4^+^ T cells enhances antitumor immunity

**Figure 3.**
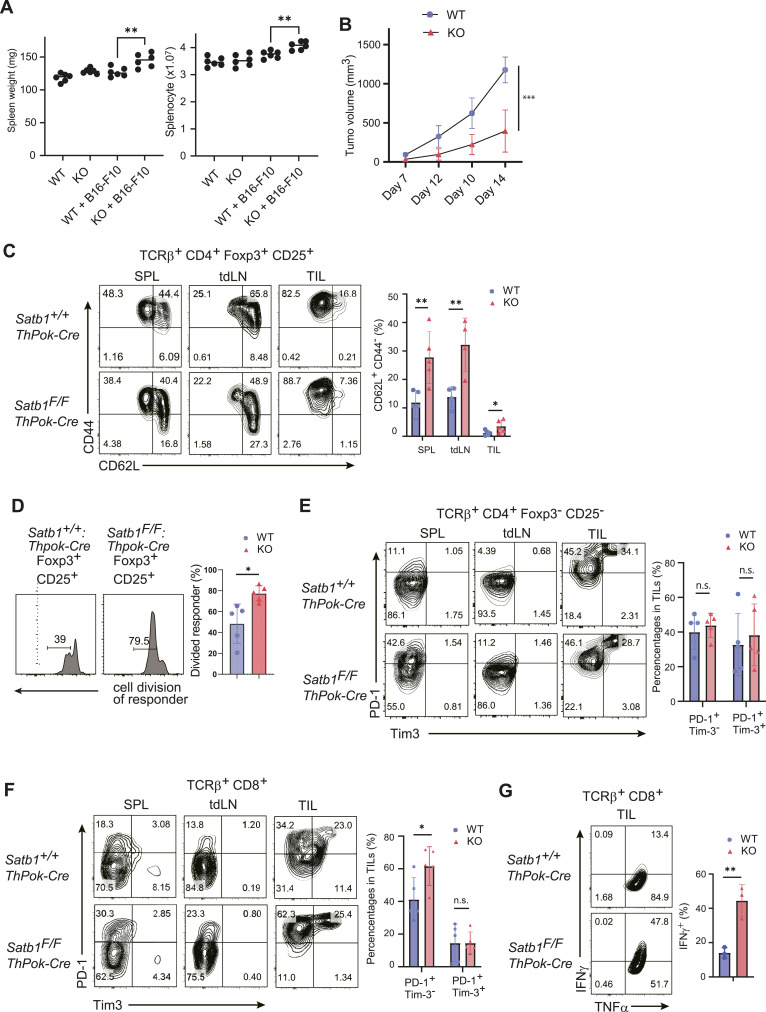
SATB1 regulates Treg functionality under tumor burden. B16-F10 melanoma cells were subcutaneously injected into hind right flanks of *WT* and *Satb1*^*F/F*^:*Thpok-cre* mice. About 2 wk later, mice were euthanized for the analysis. The summary graph shows the weights of spleens and numbers of splenocytes (A) and the tumor sizes (B) with mice with indicated genotypes (n = 7). Error bars indicate mean ± SD and each dot represents a mouse examined over at least two independent experiments. Statistical significance is measured via unpaired two-tailed *t* tests for Day 14 points. Flow cytometry analysis for CD44/CD62L, PD-1/Tim3, or IFNγ/TNFα (intracellular staining) from the splenic, tumor-draining lymph nodes, or TILs of TCRβ^+^ CD4^+^FoxP3^+^CD25^+^ (C), TCRβ^+^CD4^+^FoxP3^−^CD25^−^ (E), and TCRβ^+^CD8^+^ T cells (F) are shown. **(D)** Intratumoral Treg cells were purified and subjected to suppression assay. The suppressor and responder cell ratios were 1:1. A summary graph of duplicates from two mice is shown on the right. **(G)** IFNγ and TNFα of intratumoral CD8^+^ T cells were intracellularly stained. A summary graph of duplicates from two mice is shown on the right. One representative profile from more than three independent experiments is shown as dot plots. Numbers in the dot plots indicate the percentage of cells in each quadrant. Statistical significance is measured via unpaired two-tailed *t* tests and is presented as follows: n.s. nonsignificant, *P < 0.05, **P < 0.01, ***P < 0.001.

Given the compromised function of Tregs in the absence of SATB1, we investigated the physiological consequences in a tumor model. Fourteen days after subcutaneous inoculation with B16-F10 melanoma cells, *Satb1*^*F/F*^*:Thpok-cre* mice showed an inflammatory phenotype, including increased spleen weights and splenocyte numbers (Fig 3A), and a substantial delay in tumor growth compared with WT mice (Fig 3B). Analysis of tumor-bearing mice showed that peripheral Tregs in the spleen and tumor-draining LN (tdLN) of SATB1-deficient hosts exhibited a more naïve (CD62L^+^CD44^−^) status (Fig 3C), consistent with our observations in tumor-free mice (Fig 2E), whereas Tregs within tumor-infiltrating lymphocytes (TILs) were highly activated in both genotypes (Fig 3C). Most importantly, intratumoral Tregs isolated from SATB1-deficient mice were less suppressive in an ex vivo suppression assay (Fig 3D).

Analysis of non-Treg cells (CD4^+^FoxP3^−^CD25^−^) showed that peripheral CD4^+^ helper T cells lacking SATB1 had higher PD-1 expression (Fig 3E), indicating derepression of *Pdcd1* in the absence of SATB1, similar to the pre-tumor condition (Fig 1A). However, the ratio of PD-1^+^Tim3^+^ (exhausted) and PD-1^+^Tim3^−^ (effector) T cells was unchanged despite the derepression of *Pdcd1*. This suggests that the functionality of CD4^+^ helper T cells within TILs is not significantly affected by the absence of SATB1. The weakened Treg compartment appeared to unleash a more potent cytotoxic response; CD8^+^ T cells from the spleen and TILs of *Satb1*^*F/F*^:*Thpok-cre* mice showed a lower ratio of exhausted (PD-1^+^Tim3^+^) to effector cells and produced more IFNγ (Fig 3F and G). This is likely an indirect effect, because the *Thpok-cre* transgene strictly targets the CD4^+^ lineage. Therefore, the enhanced CD8^+^ T-cell effector function is an indirect consequence of the compromised suppressive capacity of SATB1-deficient Tregs within the tumor microenvironment. The targeted failure of Tregs to produce adequate IL-10 and maintain repression of *Lef1/Pde3b* effectively unleashes a more potent systemic cytotoxic response.

### SATB1 is necessary for proper function of Treg under tumor burden

**Figure 4.**
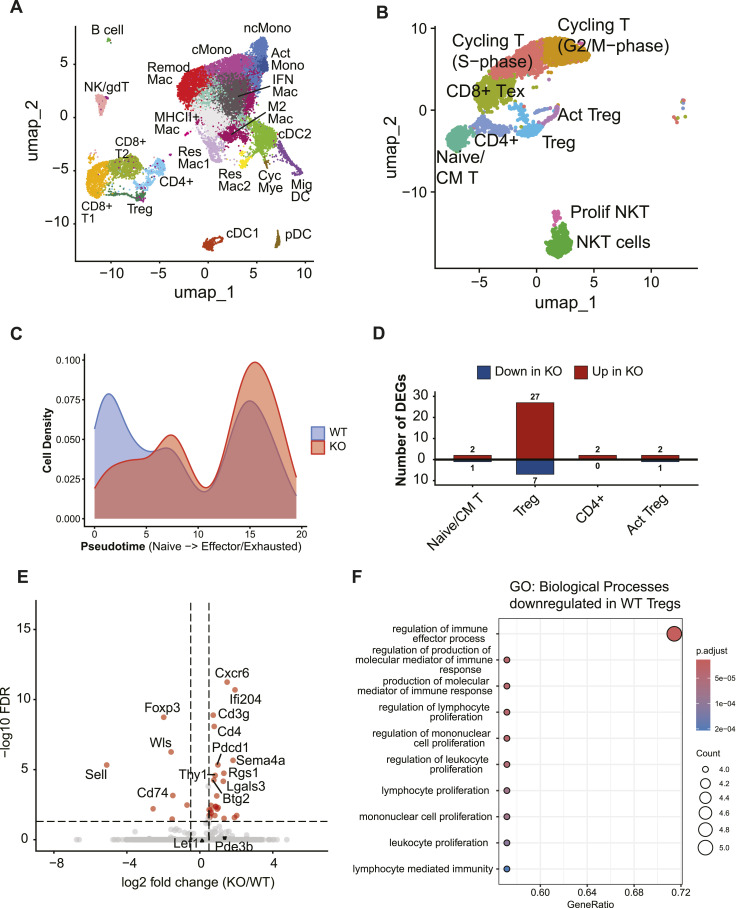
SATB1 is necessary for proper function of Treg under tumor burden. **(A)** UMAP visualization of scRNA-seq data integrating 20 distinct immune cell clusters from CD45^+^ TILs isolated from B16-F10 tumor–bearing WT and *Satb1* cKO mice. **(B)** Isolated re-clustering of T-cell populations, identifying nine distinct T-cell subpopulations. **(C)** Pseudotime trajectory analysis mapping the progression of intratumoral T cells from naïve to effector/exhausted states. **(D)** Bar graph quantifying the total number of up- and down-regulated differentially expressed genes across different T-cell subclusters in the absence of SATB1. **(E)** Volcano plot detailing the transcriptomic alterations specifically within the intratumoral Treg subpopulation, highlighting reduced *Foxp3* and increased *Pdcd1*. **(F)** Gene Ontology biological pathways significantly down-regulated in SATB1-deficient Tregs, emphasizing a reduction in immune effector regulatory processes.

**Figure S2.**
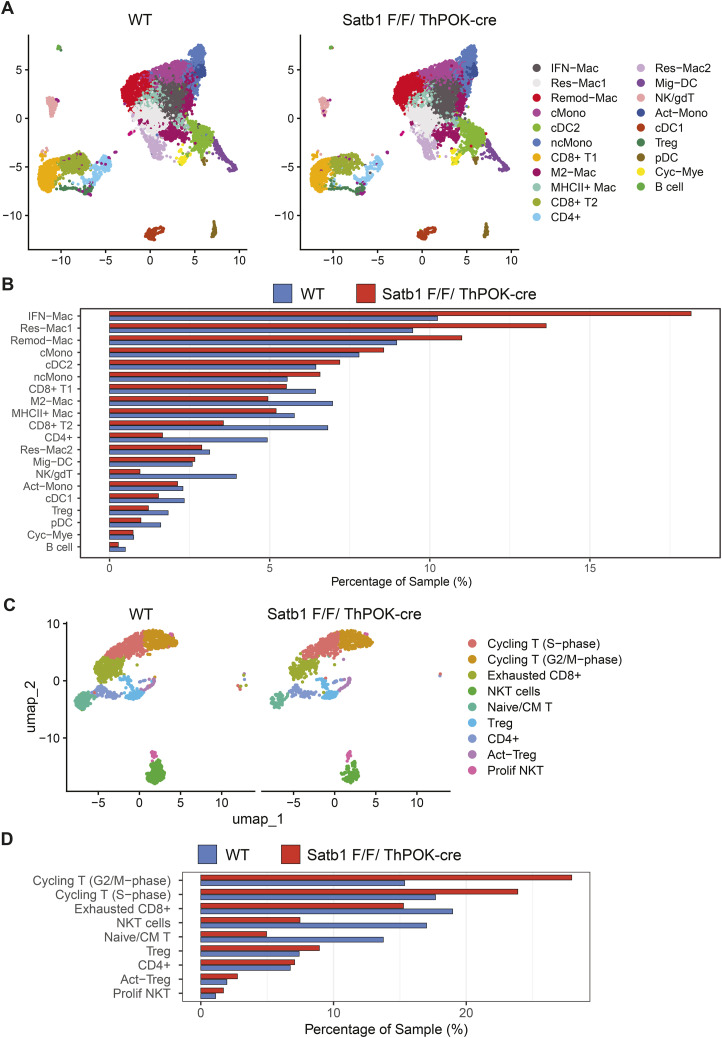
Single-cell RNA-sequencing analysis of tumor-infiltrating immune populations. **(A)** Split UMAP visualizations of all sorted CD45^+^ tumor-infiltrating lymphocytes isolated from WT and *Satb1* ^*F/F*^*:Thpok-cre* mice. **(B)** UMAP projection denoting the 20 identified major immune cell clusters across both genotypes, including various macrophage, monocyte, dendritic cell, and lymphocyte lineages. **(C)** Isolated UMAP re-clustering of T-cell subpopulations, displaying the distribution of distinct T-cell states (e.g., naive/CM T, cycling T, exhausted CD8^+^, Treg, Act-Treg) separated by genotype. **(D)** Bar graphs quantifying the relative sample percentages for the global immune cell clusters and the isolated T-cell subpopulations, highlighting the proportional shifts within the tumor microenvironment after SATB1 deletion.

To dissect the molecular mechanisms underlying the compromised suppressive function of SATB1-deficient Tregs isolated from *Thpok-cre* mice within the complex tumor microenvironment, we performed single-cell RNA sequencing (scRNA-seq) on sorted CD45^+^ TILs from B16-F10 melanoma–bearing *Satb1*^*F/F*^:*Thpok-cre* and WT mice. An integrated analysis of all immune cells from both genotypes, visualized using UMAP, revealed 20 distinct clusters corresponding to major immune lineages (Fig 4A). While the absence of SATB1 did not lead to the complete loss or emergence of any novel cell clusters (Fig S2A), it did induce notable proportional shifts between the genotypes (Fig S2B). However, several lymphoid lineages, including CD8^+^ T, CD4^+^ T, NK, γδ T, and Treg cells, exhibited reduced frequencies in *Satb1*^*F/F*^:*Thpok-cre* mice. This systemic shift suggests that SATB1 deletion within the CD4^+^ T-cell compartment exerts broad yet indirect effects on the overall antitumor immune landscape.

**Figure S3.**
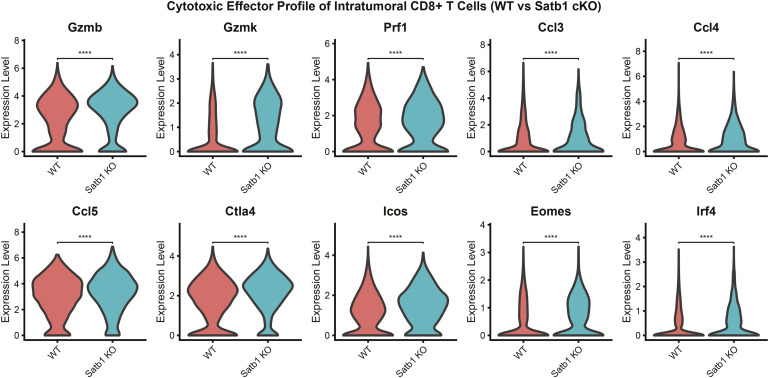
Enhanced cytotoxic and effector programs in intratumoral CD8^+^ T cells after CD4-specific SATB1 deletion. Violin plots depicting the expression levels of canonical cytotoxic molecules (*Gzmb*, *Gzmk*, and *Prf1*), and other canonical markers for T-cell cytotoxicity queried from the scRNA-seq dataset. Data compares intratumoral CD8^+^ T cells isolated from WT and *Satb1F/F*:*Thpok-cre* melanoma–bearing mice. Statistical significance was determined using a Wilcoxon rank-sum test (****P < 0.0001).

To gain higher resolution into the T-cell compartment, we isolated and reclustered these cells, identifying nine distinct T-cell subpopulations (Fig 4B). Similar to the global immune landscape, *Thpok-cre*–mediated SATB1 deficiency in T cells did not fundamentally alter the presence of these subclusters (Fig S2C), but it did skew their relative proportions (Fig S2D). Notably, cycling CD8^+^ T cells were increased, whereas exhausted T cells were decreased, a profile consistent with the robust antitumor response and reduced tumor sizes observed in these mice. Furthermore, pseudotime trajectory analysis revealed that intratumoral T cells preferentially progressed from naïve toward effector/exhausted states in the absence of SATB1 (Fig 4C). This trajectory shift further supports the premise that SATB1 expression in CD4^+^ T cells plays a substantial role in modulating global T-cell antitumor immunity. To comprehensively profile this increased cytotoxic response, we examined core cytotoxic mediators (including *Gzmb*, *Gzmk*, and *Prf1*) and canonical genes related to T-cell cytotoxicity (Fig S3), which further underscored an intensely active effector state within the tumor microenvironment of CD8^+^ T cells in the absence of SATB1 in CD4^+^ T cells.

Despite these pronounced functional phenotypes, global transcriptomic alterations within most CD4^+^ T-cell subclusters were surprisingly mild. However, distinct and focused changes were observed specifically within the Treg subpopulation (Fig 4D), aligning with our earlier findings that SATB1 plays a more critical role in Tregs than in conventional CD4^+^ T cells (Fig 3E). Within the tumor microenvironment, SATB1-deficient Tregs displayed a reduction in *Foxp3* expression coupled with increased *Pdcd1* expression (Fig 4E). Only 30 genes were significantly altered in the knockout Tregs; consequently, only a single Gene Ontology biological process, pertaining to immune effector regulatory function, was significantly down-regulated (Fig 4F).

These highly targeted transcriptomic shifts indicate that SATB1 in FoxP3^+^CD25^+^ Tregs from *Thpok-cr*e mice is not strictly required for the differentiation or maintenance of distinct intratumoral Treg subsets. Collectively, our single-cell data illustrate that rather than broadly dictating the structural identity or heterogeneity of intratumoral Tregs, SATB1 acts as an indispensable fine-tuner.

## Discussion

Understanding the distinct functions of the genome organizer SATB1 in peripheral T cells has historically been constrained by the severe developmental defects observed in early-stage conditional knockout models. By leveraging the *Thpok-cre* driver to specifically ablate *Satb1* after CD4 lineage commitment, our study unmasks a dual requirement for SATB1 in maintaining peripheral immune homeostasis. We demonstrate that SATB1 is strictly required to repress *Foxp3* and *Pdcd1* in conventional CD4^+^ T cells. However, the aberrant derepression of FoxP3 in these conventional cells is insufficient to drive a complete regulatory transcriptomic program or confer suppressive capabilities. This suggests that merely turning on *Foxp3* in a mature CD4^+^ T cell lacks the necessary foundational chromatin architecture required to execute a suppressive program.

Furthermore, we illustrate the functional significance of SATB1 between CD4 lineage commitment and *Foxp3* expression by using the *Thpok-cre* driver. We found that Tregs generated under this condition exhibit functional impairments. This finding highlights a strict temporal requirement for SATB1-mediated genome organization between CD4 lineage commitment and the onset of FoxP3 expression. During this time window, SATB1-mediated two-tiered chromatin organization linked to cell-type–specific gene expression (33) may mature to fully establish the foundational transcriptional programming required for Tregs. Therefore, we propose that mature Treg suppressive activity may become SATB1-independent only if this foundational programming is allowed to complete.

This reveals a stark dichotomy: in conventional T cells, SATB1 acts primarily as a repressor, whereas in *bona fide* Tregs, it functions as an essential co-factor to maintain suppressive fitness. SATB1-deficient Tregs exhibit reduced IL-10 production and a profound inability to suppress responder T cells, driven by a targeted failure to silence key regulators such as *Lef1* and *Pde3b*. Physiologically, this Treg-specific functional defect significantly enhances antitumor immunity in a melanoma model, unleashing a more potent cytotoxic CD8^+^ T-cell response.

Single-cell transcriptomics confirm that SATB1 acts as a critical fine-tuner of Treg fitness rather than a broad determinant of structural heterogeneity. This role extends to the CD8^+^ T-cell compartment, where SATB1 is similarly required to sustain long-term functional fitness under chronic stress (23, 34). Taken together, a unified paradigm emerges: SATB1 serves as a broadly essential guardian of mature T cells, required to preserve lineage fidelity and functional fitness under chronic immunological stress. Targeting the SATB1-dependent regulatory axis may therefore offer a highly selective avenue for relieving Treg-mediated immunosuppression in cancer immunotherapy.

## Materials and Methods

### Mice

*Satb1*^*Venus*^ (Ichiro Taniuchi lab) (4), *FoxP3*^*GFP*^ (Marie Malissen lab) (24), *Satb1*^*F/F*^ (Terumi Kohwi-Shigematsu lab) (11), *Thpok-cre* (Ichiro Taniuchi lab) (18) mice have been previously described. Mice used in this study were 8–12 wk on average and were mixed sexes unless otherwise specified. Mice were euthanized by suffocation with CO_2_ under anesthesia or cervical dislocation. All mice were maintained in the specific pathogen-free animal facility at the RIKEN IMS and Nagoya University, and all animal procedures were in accordance with institutional guidelines for animal care and with the protocol (28-017) approved by the Institutional Animal Care and Use Committee of RIKEN Yokohama Branch as well as by the Institutional Animal Experimental Committee of Nagoya University.

### Cell cultures

T cells were enriched by magnetic cell isolation (Miltenyi Biotec) from spleens or lymph nodes and sorted by FACS Aria (BD Biosciences). Purified T cells were cultured in custom-ordered Dulbecco’s Modified Eagle Media (D-MEM, KOHJIN BIO) supplemented with 10% fetal bovine serum (Hyclone). For typical experiments involving in vitro culture, 2.0 × 10^5^ T cells were stimulated in 96-well plates through immobilized 2 μg/ml anti-CD3e antibody (553058; BD Biosciences) with 2 μg/ml soluble anti-CD28 antibody (553295; BD Biosciences) for 2 d. Stimulated T cells were further maintained with 40  U/ml rmIL-2 (402-ML; R&D Systems). For exhaustion conditions, cells were maintained on fresh plates immobilized with anti-CD3e antibody and soluble anti-CD28 antibody for three more days with rmIL-2 supplementation. B16-F10 cell line was maintained in D-MEM medium (GIBCO) supplemented with 10% FBS. All cell culture media were supplemented with 100  U/ml penicillin and 100 μg/ml streptomycin.

### Treg suppression assay

CD4^+^CD25^−^ or CD8^+^CD25^−^ responder T cells were purified from Ly5.1 WT mice using FACSAria III and stained with 10 μm Cell proliferation Dye eFluor 450 (eBioscience) or CytoTell Blue (AAT Bioquest). The labeled responder T cells (5 × 10^4^ cells/well) were cultured alone or together with CD4^+^Foxp3^+^CD25^+^, CD4^+^Foxp3^+^CD25^−^, or CD4^+^Foxp3^-^CD25^−^ suppressor cells purified from SATB1-deficient or WT mice (Ly5.2) at different ratios in 96-well round-bottom plates. The cells were stimulated with 0.5 μg/mL anti-CD3ε antibody in the presence of irradiated (20 Gy) T cell-depleted splenocytes (2 × 10^5^ cells/well), which were prepared from Ly5.2 WT mice by MACS magnetic cell separation (Miltenyi Biotec). At 3 d after stimulation, the cell proliferation dye dilution in Ly5.1^+^ cells was analyzed by flow cytometry.

### RNA-seq and bioinformatic analysis

Purified mRNAs were used for library construction with the NEBNext Ultra II RNA Library Prep Kit for Illumina (NEB) according to the manufacturer’s protocol. The library was quantified by KAPA Library Quantification Kit (KAPA Biosystems), and sequenced by HiSeq 2000/2500 platform (Illumina). Sequencing results were aligned to the mouse reference genome (mm10) using HISAT2 (version 2.1.0) with standard parameters. Read counts were obtained by HTSeq (version 0.11.0) and read counts were analyzed by the Bioconductor package EdgeR (version 3.14).

### scRNA-seq and bioinformatic analysis

Tumor tissue was treated with BD Horizon Dri Tissue and Tumor Dissociation Reagent (BD Biosciences), and CD45^+^ cells were sorted (BD Symphony S6). Sorted cells were fixed by Chromium Next GEM Single Cell Fixed RNA Sample Preparation Kit (10x Genomics) and frozen at −80°C until ready. 1,000,000 cells were loaded onto Chromium X and 12,000–14,000 cells per sample were captured for library preparation by Chromium Fixed RNA Kit, Mouse Transcriptome (10x Genomics). Sequence was read by HiSeq 4000 and aligned to the mm10 mouse genome using cellranger, which was further analyzed using the Seurat package. Cells with <1,000 detected genes or a mitochondrial read percentage >5 were discarded. Data were normalized, scaled, merged, and integrated with three samples. Highly variable genes were identified with the *vst* protocol, which were used to construct principal components. Clusters were withdrawn using the top 20 principal components and visualized using dimensional reduction algorithm UMAP.

Pseudotime trajectory inference was performed on the subsetted T-cell populations using the *Slingshot* package in R. Trajectory lineages were constructed using the UMAP dimensional reduction embeddings and the annotated cell type clusters. To map the developmental progression, the trajectory was directionally anchored by explicitly designating the naive/central memory T-cell cluster as the starting root node. The calculated pseudotime values from the primary lineage, representing the dominant progression path from naive toward effector and exhausted states, were extracted and added to the original Seurat metadata.

### Flow cytometry and antibodies

Cells were prepared from the thymocytes, spleen, and lymph nodes by standard procedures. Briefly, a single-cell suspension was prepared by passing through 70-μm cell strainers and lysing red blood cells. Cells were stained with the following antibodies purchased from BD Bioscience, BioLegend, or Thermo Fisher Scientific: B220 (RA3-6B2k), CD4 (RM4-5), CD8β (YTS156.7.7), CD25 (PC61), CD44 (IM7), CD62L (MEL-14), CD69 (H1.2F3), FoxP3 (FJK-16s), IFNγ (XMG1.2), PD-1 (RMP1-30), TCRβ (H57-597), and Tim3 (RMT3-23), which were used at 0.5 μg/ml. Antibodies including B220, CD4, and CD8β were used for lineage gating. For intracellular staining, cell suspensions were fixed and permeabilized at the same time by BD Perm/Fix solutions (BD Biosciences). Multicolor flow cytometry analysis was performed using a FACSCanto II/Symphony A3/S6 (BD Biosciences) and data were analyzed using FlowJo version 10 (Tree Star) software.

### B16 tumor model

For the tumor-burden model, WT (C57BL/6) mice were subcutaneously (s.c.) inoculated with 5 × 10^5^ B16-F10 cells. Tumor growth was monitored by measuring three perpendicular diameters, and tumor volume was calculated according to the formula V = L × W^2^ × 0.52, where V is the volume, L is the length, and W is the width.

### Statistical analysis

The individual dots on the summary graphs indicate independent mice analyzed, unless otherwise specified. Statistical parameters are indicated in the figure legends. All bar and dot graphs show mean values ± SD. Statistical analysis was performed using an unpaired two-tailed *t* test in GraphPad Prism Version 10. The *P*-value of <0.05 was considered statistically significant.

## Supplementary Material

Reviewer comments

## Data Availability

The raw data for RNA-seq have been deposited in the GEO database under the accession code GSE190915 super series.
