# Supplementary material for: SATB1 preserves CD4+ T-cell fidelity and establishes Treg function in antitumor immunity
Source: Life Sci Alliance. 2026 Jul 14;9(10):e202603763. doi: 10.26508/lsa.202603763 (PMC13370069; doi:10.26508/lsa.202603763)
Supplement: Reviewer comments [file LSA-2026-03763_review_history.pdf]

SATB1 preserves $CD4^+$ T cell fidelity and establishes Treg function in anti-tumor immunity

Wooseok Seo, Chengcheng Zou, Kanako Shimizu, Ruka Setoguchi, Kiyokazu Kakugawa, Terumi Kohwi-Shigematsu, Shohei Hori, Shin-ichiro Fujii, Hiroyoshi Nishikawa, and Ichiro Taniuchi
DOI: https://doi.org/10.26508/lsa.202603763

Corresponding author(s): Wooseok Seo, Kyoto University and Ichiro Taniuchi, RIKEN Center for Integrative Medical Sciences

Review Timeline:

Submission Date:	2026-05-10
Editorial Decision:	2026-05-12
Revision Received:	2026-06-07
Editorial Decision:	2026-06-30
Revision Received:	2026-07-01
Accepted:	2026-07-02

Scientific Editor: Tim Fessenden

Transaction Report:

(Note: With the exception of the correction of typographical or spelling errors that could be a source of ambiguity, letters and reports are not edited. The original formatting of letters and referee reports may not be reflected in this compilation.)

Please note that the manuscript was previously reviewed at another journal and the reports were taken into account in the decision-making process at *Life Science Alliance*. Since the original reviews are not subject to Life Science Alliance's transparent review process policy, the reports and author response cannot be published.

May 12, 2026

Re: Life Science Alliance manuscript #LSA-2026-03763-T

Dr. Wooseok Seo
Kyoto University
Graduate School of Medicine
Yoshida-Konoe-cho, Sakyo-ku
Kyoto 606-8501
Japan

Dear Dr. Seo,

Thank you for transferring your manuscript entitled "SATB1 preserves CD4 $^{+}$ T cell lineage fidelity and enforces Treg suppressive function to regulate anti-tumor immunity" to Life Science Alliance. This work offers important new findings on epigenetic regulation of CD4 T cell identity and function, some of which contrast with prior claims on SATB1, and would be appreciated by LSA readers. We also acknowledge your remarks on the concerns raised by peer reviewers from another journal. In light of the reviewer comments and our interest in these observations, we are pleased to offer consideration of this manuscript at LSA.

I would be happy to discuss the revision in more detail via email or phone/videoconferencing. Please let me know which option you prefer, if any.

To upload the revised version of your manuscript, please log in to your account: https://lsa.msubmit.net/cgi-bin/main.plex

You will be guided to complete the submission of your revised manuscript and to fill in all necessary information. Please get in touch in case you do not know or remember your login name.

The typical timeframe for revisions is three months. Please note that papers are generally considered through only one revision cycle, so strong support from the referees on the revised version is needed for acceptance.

While you are revising your manuscript, please also attend to the below editorial points to help expedite the publication of your manuscript. Please direct any editorial questions to the journal office. When submitting the revision, please include a letter addressing the reviewers' comments point by point.

Thank you for this interesting contribution to Life Science Alliance. We hope that the comments below will prove constructive as your work progresses, and we are looking forward to receiving your revised manuscript.

Sincerely,

Tim Fessenden
Scientific Editor
Life Science Alliance

A. THESE ITEMS ARE REQUIRED FOR REVISIONS

-- A letter addressing the reviewers' comments point by point.

-- An editable version of the final text (.DOC or .DOCX) is needed for copyediting (no PDFs).

-- High-resolution figure, supplementary figure and video files uploaded as individual files: See our detailed guidelines for preparing your production-ready images, https://www.life-science-alliance.org/authors

-- Summary blurb (enter in submission system): A short text summarizing in a single sentence the study (max. 200 characters including spaces). This text is used in conjunction with the titles of papers, hence should be informative and complementary to the title and running title. It should describe the context and significance of the findings for a general readership; it should be written in the present tense and refer to the work in the third person. Author names should not be mentioned.

-- By submitting a revision, you attest that you are aware of our payment policies found here: https://www.life-science-alliance.org/copyright-license-fee

B. MANUSCRIPT ORGANIZATION AND FORMATTING:

Full guidelines are available on our Instructions for Authors page, https://www.life-science-alliance.org/authors

We encourage our authors to provide original source data, particularly uncropped/-processed electrophoretic blots and spreadsheets for the main figures of the manuscript. If you would like to add source data, we would welcome one PDF/Excel-file per figure for this information. These files will be linked as supplementary "Source Data" files.

IMPORTANT: It is Life Science Alliance policy that if requested, original data images must be made available. Failure to provide original images upon request will result in unavoidable delays in publication. Please ensure that you have access to all original microscopy and blot data images before submitting your revision.

June 30, 2026

RE: Life Science Alliance Manuscript #LSA-2026-03763-TR

Dr. Wooseok Seo
Kyoto University
Graduate School of Medicine
Yoshida-Konoe-cho, Sakyo-ku
Kyoto 606-8501
Japan

Dear Dr. Seo,

Thank you for submitting your revised manuscript entitled "SATB1 preserves CD4⁺ T cell fidelity and establishes Treg function in anti-tumor immunity". In accordance with our prior correspondence, we returned this work to the original Reviewer 1 whose comments are below.

As you will see, this reviewer commends the revised work for resolving some of their concerns and they point out a few remaining issues to be resolved.

MANUSCRIPT ORGANIZATION AND FORMATTING:

To avoid unnecessary delays in the acceptance and publication of your paper, please read the following information carefully. Full guidelines are available on our Instructions for Authors page, https://www.life-science-alliance.org/authors

Submission of a paper that does not conform to Life Science Alliance guidelines will delay the acceptance of your manuscript.

-It is recommended to exclude figures from the manuscript text and upload them separately.
-Please upload your main and supplementary figures as single files.
-Please add the X and Bluesky handles of your host institute/organization, as well as your own, and/or one of the authors, in our system.
-Please upload a clean manuscript file. The highlighted version (with included changes) should be uploaded as a "Related Manuscript file."
-Please rename "Competing interests" to "Conflict of Interest."
-Please add your main and supplementary figure legends to the main manuscript text after the references section and remove them from the figures.
-There are call-outs in the manuscript text for figure S1A-D...we believe this should be for fig/ S2A-D. Could you please check and correct?
-LSA does not permit citation of "data not shown," "manuscript in preparation," "manuscript submitted," etc., in any section of the manuscript (e.g., line 195). Please either provide the analysis demonstrating no significant pathways, or remove this statement.
-Please indicate the sources for all mouse strains. Please provide details on the plasmids used to generate retrovirus, and primers used for QPCR. Finally please ensure full details on sequencing analysis are provided, including pseudotime analysis.
-Please add y axis labels to Figure 1 D (right panel).

We encourage our authors to provide original source data, particularly uncropped/-processed electrophoretic blots and spreadsheets for the main figures of the manuscript. If you would like to add source data, we would welcome one PDF/Excel-file per figure for this information. These files will be linked as supplementary "Source Data" files.

It is Life Science Alliance policy that if requested, original data images must be made available to the editors. Failure to provide original images upon request will result in unavoidable delays in publication. Please ensure that you have access to all original data images prior to final submission.

If you are planning a press release on your work, please inform us immediately to allow informing our production team and scheduling a release date.

We welcome submissions of potential cover images for the issue of LSA in which your work would appear. If you have high quality images associated with this work, please feel free to email these, with a caption, to the journal office.

LSA encourages authors to provide a 30-60 second video where the study is briefly explained. These videos will be appear embedded with the manuscript online at Life Science Alliance, and on social media to promote the published paper and authors (for examples, see https://docs.google.com/document/d/1-UWCfbE4pGcDdcgzcmiuJl2XMBJnxKYeqRvLLrLSo8s/edit?usp=sharing). Corresponding or first-authors are welcome to submit the video. Please submit only one video per manuscript. The video can be emailed to contact@life-science-alliance.org

FINAL FILES:

To upload the final version of your manuscript, please log in to your account: https://lsa.msubmit.net/cgi-bin/main.plex
You will be guided to complete the submission of your revised manuscript and to fill in all necessary information. Please get in touch in case you do not know or remember your login name.

The following items are required for acceptance.

-- An editable version of the final text (.DOC or .DOCX) is needed for copyediting (no PDFs).

-- High-resolution figure, supplementary figure and video files uploaded as individual files: See our detailed guidelines for preparing your production-ready images, https://www.life-science-alliance.org/authors

-- Summary blurb (enter in submission system): A short text summarizing in a single sentence the study (max. 200 characters including spaces). This text is used in conjunction with the titles of papers, hence should be informative and complementary to the title. It should describe the context and significance of the findings for a general readership; it should be written in the present tense and refer to the work in the third person. Author names should not be mentioned.

The license to publish form must be signed before your manuscript can be sent to production. A link to the license to publish form will be available to the corresponding author only. Please take a moment to check your funder requirements.

Reviews, decision letters, and point-by-point responses associated with peer-review at Life Science Alliance will be published, alongside the manuscript. If you do want to opt out of having the reviewer reports and your point-by-point responses displayed, please let us know immediately.

Thank you for your attention to these final processing requirements. Please revise and format the manuscript and upload materials as soon as you are able.

Thank you for this interesting contribution to the literature. We look forward to publishing your paper in Life Science Alliance.

Sincerely,

Tim Fessenden
Scientific Editor
Life Science Alliance

July 2, 2026

RE: Life Science Alliance Manuscript #LSA-2026-03763-TRR

Dr. Wooseok Seo
Kyoto University
Graduate School of Medicine
Yoshida-Konoe-cho, Sakyo-ku
Kyoto 606-8501
Japan

Dear Dr. Seo,

Thank you for submitting your Research Article entitled "SATB1 preserves $CD4^+$ T cell fidelity and establishes Treg function in anti-tumor immunity". We note the resolution of the remaining reviewer points and the formatting issues we requested you address. It is a pleasure to let you know that your manuscript is now accepted for publication in Life Science Alliance. Congratulations on this interesting work.

We kindly request the resolution of a final formatting issue during the proofing and copyediting process. As noted in our prior decision letter, the quantification shown in Figure 1D is missing numerical labels on the y axis (percent PD-1+ cells). During the proofing process, please replace Figure 1 with a corrected figure file that includes these labels.

The final published version of your manuscript will be deposited by us to PubMed Central upon publication.

Your article will be published open access upon publication under a CC-BY license.

Reviews, decision letters, and point-by-point responses associated with peer-review at Life Science Alliance will be published, alongside the manuscript. If you do want to opt out of having the reviewer reports and your point-by-point responses displayed, please let us know immediately.

IMPORTANT: If you will be unreachable at any time, please provide us with the email address of an alternate author. Failure to respond to routine queries may lead to unavoidable delays in publication.

Scheduling details will be available from our production department. You will receive proofs shortly before the publication date. Only essential corrections can be made at the proof stage so if there are any minor final changes you wish to make to the manuscript, please let the journal office know now.

DISTRIBUTION OF MATERIALS:
Authors are required to distribute freely any materials used in experiments published in Life Science Alliance. Authors are encouraged to deposit materials used in their studies to the appropriate repositories for distribution to researchers.

You can contact the journal office with any questions, contact@life-science-alliance.org

Again, congratulations on a very nice paper. I hope you found the review process to be constructive and are pleased with how the manuscript was handled editorially. We look forward to future exciting submissions from your lab.

Sincerely,

Tim Fessenden
Scientific Editor
Life Science Alliance
